# Polydrug use in Australian 12-14 year olds from 2006 to 2017: an examination of drug use profiles, emotional control problems, and family relationship characteristics

**DOI:** 10.1080/00049530.2023.2174705

**Published:** 2023-02-19

**Authors:** Adrian B. Kelly, Andrew Munnings, Xiang Zhao, Bosco Rowland, Kristin R. Laurens, Marilyn Campbell, Joanne Williams, Jen A. Bailey, Callula Killingly, Julie Abimanyi-Ochom, Peter Kremer, John W. Toumbourou

**Affiliations:** a Centre for Inclusive Education, Queensland University of Technology, Brisbane, Australia; b School of Psychology and Counselling, Queensland University of Technology, Brisbane, Australia; c Centre for Child Health and Well-being (Childhood Adversity, Mental Health, and Resilience Theme), Queensland University of Technology, Brisbane, Australia; d School of Law, Psychology and Social Work, Örebro University, Örebro, Sweden; e School of Psychology and Centre for Social and Early Emotional Development, Deakin University, Melbourne, Australia; f Faculty of Medicine and the Eastern Health Clinical School, Monash University Melbourne, Australia; g School of Early Childhood and Inclusive Education, Queensland University of Technology, Brisbane, Australia; h School of Health, Swinburne University of Technology, Melbourne, Australia; i Social Developmental Research Group, University of Washington, Seattle, WA, USA; j School of Health and Social Development, Deakin University, Melbourne, Australia; k School of Exercise and Nutrition Sciences and Centre for Sport Research, Deakin University, Melbourne, Australia

**Keywords:** Adolescent, polydrug use, family relationships, family conflict, academic performance, emotional control

## Abstract

**Objective:**

This study examined the nature and prevalence of polydrug use in 12–14 year old Australians.

**Method:**

Three Australian school surveys (2006, *n*=4091; 2009, *n*=5635; 2017, *n*=1539; age 12–14 years) spanning 11 years were used. Substances included alcohol, tobacco, cannabis, inhalant, and other illicit substances. Risk factors included depressed mood, low emotional control, poor family management and conflict, and academic performance. Latent class analysis was used to discern classes. Regression analyses were used to test the association of risk factors with classes.

**Results:**

Consistent across surveys, there was a class of adolescents who engaged in wide-ranging polydrug use, with prevalences ranging from 0.44% (2006) to 1.78% (2017). Emotional control problems, low academic performance, and poor family management were elevated in the polydrug class.

**Conclusion:**

A small proportion of 12–14-year-old adolescents engage in polydrug use. Interventions focusing on family risks and emotional control problems may be beneficial.

## Key points

**What is already known about this topic**

1. In Australia, adolescents have generally reduced their use of alcohol and tobacco over recent decades.
2. Most research is based on patterns of use of single substances in mid-to-late adolescence, but we know that a significant proportion of older Australian adolescents engage in polydrug use.
3. Family relationship quality has been associated with drug use amongst older adolescents and young adults but may have an especially significant association with polydrug use amongst younger adolescents given key biopsychosocial transitions occurring around this age.

**What this research adds:**

1. A small but meaningful proportion of Australian 12–14-year-olds engage in polydrug use.
2. The nature of polydrug use amongst young Australian adolescents has shifted since 2006, with profiles showing decreased tobacco use and continuing challenges in addressing alcohol, cannabis and inhalant use amongst young adolescents. This group also reported poor family management, poor emotional control, and academic failure.
3. The results highlight the importance of detection and targeted early intervention for a subgroup of young adolescents who may have developed risky drug use patterns across the transition to high school.

## Introduction

Amongst Australian adolescents 14–17 years of age, there have been significant downward trends over recent decades in the use of alcohol and certain other drugs. Since 1998, heavy episodic alcohol use (5+ drinks/day) and weekly alcohol use have steadily decreased and there has been a relatively steady increase in the prevalence of alcohol abstinence (Kelly et al., 2016). There have been significant declines in tobacco initiation among 14+ year olds (Australian Institute of Health and Welfare, 2020). From 2001 to 2016, there were decreases in the lifetime prevalence and recent use (last 12 months) of cannabis amongst 15–19 year olds (from approximately 34% in 2001 to 17% in 2016) (Australian Institute of Health and Welfare, 2020). Amongst 14–19 year olds, there were substantial drops in recent use of meth/amphetamine from 2001 to 2007 (use for non-medical purposes), a levelling out of recent use until 2013, and further decreases in recent use by 2016 (Australian Institute of Health and Welfare, 2020). While downward trends in the recent use of specific substances by middle to later aged adolescents are positive from a public health perspective, these trends provide little or no information on adolescents who are polydrug users (recent users of 2+ drug types).

Comparatively few studies are available in Australia and internationally that quantify polydrug use amongst young adolescents (12–14 years of age) (see Table S1 for details of available studies). Amongst older American adolescents, polydrug use is reported by 1.7% to 2% of students (Banks et al., 2020). Connell et al. (2010) found a class of users that comprised 10% of the sample and which reported recent alcohol, tobacco, cannabis, and lifetime use of other illicit drugs, inhalants, and nonmedical use of prescribed medicines (NUPM). In one of the few nationally representative American studies of polydrug use amongst Grade 8 students, 9% reported a high probability of using any of 5 substances (e-cigarettes, tobacco, cannabis, NUPM and alcohol) (Miech et al., 2016). In Australia, polydrug use (primarily alcohol, tobacco, cannabis) is reported by 4.7% of 13.5 year olds in the prior month (Chan et al., 2016) and these rates are higher (8.2%) in an older sample of 14.3 year olds (Chan et al., 2017). Amongst Swedish Grade 9 and 11 students reporting any illicit drug use, 7% reported polydrug use (Miech et al., 2016). Across 25 European countries, 4.7% of adolescents (mean age 13.9 years) report polydrug use in the past month (Göbel et al., 2016). In summary, past month prevalence of polydrug use in the past month for middle adolescents ranges from 4 to 9%.

Rates of past year/lifetime polydrug use show large variations, primarily because of differences in sampling procedures. Around 20.3% of adolescents (12–17 years old) were classified as polydrug users (A. White et al., 2013). Specifically, 18.3% were limited range polydrug users (alcohol, tobacco and cannabis) and 2% were extended polydrug users (these plus painkillers, ecstasy, amphetamine). Research from Michigan (US) indicates that 4.6% of students had a high probability of polydrug use in the past 12 months (Cranford et al., 2013). In a young Brazilian adolescent sample (mean age 12.6 years) recruited for a prevention program and based on a restricted range of drug types, 1.8% of adolescents were classed as polydrug users (Valente et al., 2017). In a Thai sample, 11% of 12- to 15- year-olds reported past year alcohol and tobacco use and high-risk behaviours like fighting and carrying a weapon and a further 0.6% reported high-risk behaviours and illicit drug use in the past year (Assanangkornchai et al., 2018). In summary, the rates of adolescent past year/lifetime polydrug use vary from 1.8% to 24.6%.

The overall aim of this study was to examine three Australian population surveys to identify substance use subgroups (“classes”) amongst early adolescents, and to examine variations across these subgroups on depressed mood, emotion regulation problems, family management practices and academic performance. Establishing the prevalence and patterns of polydrug use amongst early adolescents is important because early onset of substance use is more strongly associated with adult drug and alcohol problems than late onset of substance use (Kelly et al., 2019), drug use is associated with great neuropsychological harm for adolescents than for adults (Lubman et al., 2016; Silveri et al., 2016; Squeglia & Gray, 2016), and simple prevention messages (e.g., alcohol or tobacco focused universal prevention) may not be useful or relevant to adolescents using multiple drugs (Kelly et al., 2019; Masterman & Kelly, 2003). An examination of the association of family relationship problems (conflict, supervision/management problems) may also inform the focus and timing of family oriented interventions.

## Materials and methods

### Sample

#### 2006 survey

The 2006 study (the *Healthy Neighbourhoods Study*) involved 7,866 adolescents (52.6% female) from 231 Australian schools (from the States of Victoria, Queensland, and Western Australia). The community sampling frame consisted of Statistical Local Areas (ABS, 2009) with greater than 17,000 inhabitants. These Statistical Local Areas were stratified into quartiles of socioeconomic disadvantage based on Socio-Economic Indexes for Areas (SEIFA), which indexes the average income and employment status for each residential postcode in Australia (ABS, 2009). Eligible communities were randomly selected from SEIFA quartiles to represent State distributions of advantage/disadvantage as well as urban and nonurban locations. A total of 164 primary and 82 secondary schools across all communities were randomly selected. Of the schools invited to participate, 83% (*n* = 443) responded, and of these, 52% agreed to participate (59% and 43% at Grade 6 and 8 levels, respectively). Students participated only if written parental consent was obtained (67% response rate). The initial sample consisted of 7,866 adolescents, with 4,091 adolescents aged between 12 and 14 years at the time of survey (*M* = 12.24; *SD* = 0.44) included in the present study. 50.2% of this sample were male.

#### 2009 survey

The 2009 sample was recruited as part of the HOWRU survey, which used a similar procedure to the 2006 study but was conducted only in Victoria and used passive parental consent. Data collection involved a two-stage sampling strategy. In the first stage, all schools in Victoria, Australia, were stratified into local government areas or school regions; schools were randomly selected from each strata based on a probability proportional to each community’s grade-level size. Overall, 220 schools from Government, Independent, and Catholic education sectors took part. In the second stage, one class from each of Grades 7, 9, and 11 at each school was randomly selected. Passive informed consent from parents was conducted for the majority of schools; however, active parent consent was required by some Catholic schools. Initially, 13501 students from Grades 7, 9, and 11 were approached, among whom 37 students declined to participate (0.3%) and 739 parents declined to provide consent (5.5%). Of the remaining participants, 2,047 were absent on the day of survey. The final sample consisted of 10,678 students (79.1% of students initially approached). Participants for this study were included if they were between 12 and 14 years of age (*n* = 5,470). The mean age of this cohort was 12.5 years (*SD* = 0.64) and 50.0% of this sample were male.

#### 2017 survey

The 2017 study recruited participants from 56 secondary schools across three Australian states (Victoria, Queensland and Western Australia) and utilised the 28 communities that initially participated in the 2006 study. The community sampling frame was identical to that in the earlier reported 2006 (Healthy Neighbourhoods) Study. The final sample of 12–14-year-old students was 1,539 adolescents with a mean age of 13.0 years (SD = 0.84), and 46.3% of this sample were male.

All procedures followed were in accordance with the ethical standards of the responsible committee on human experimentation (institutional and national) and with the Helsinki Declaration of 1975, as revised in 2000. Active parental consent was obtained for adolescent participants in the 2006 and 2017 Surveys and these surveys were approved by the University of Melbourne Human Research Ethics Committee and the Deakin University Human Research Ethics Committee respectively. Passive parental consent was used for adolescent participants in the 2009 Survey and this Survey was approved by the University of Melbourne Human Research Ethics Committee and the Victorian Department of Education.

### Measures

The measures used in the three surveys were based on the *Communities That Care Youth Survey* (CTC). This is an epidemiological assessment instrument developed in the United States (Arthur et al., 2002) and adapted for Australian youth (McMorris et al., 1998-2017), with demonstrated reliability and validity (Kelly et al., 2011). The survey takes approximately 45 minutes for a participant to complete.

#### Substance use

The following items assessed lifetime use of a range of substances, each according to a five-point response scale (*1* “never”, *2* “1 or 2 times”, *3* “3 to 5 times”, *4* “6 to 9 times”, *5* “10 or more times”), where higher scores indicating increased frequency of use. *Alcohol*: “In your lifetime have you had more than just a few sips of an alcoholic beverage (like beer, wine or spirits)?”; *Tobacco*: “In your lifetime have you ever smoked cigarettes?”; *Cannabis*: “In your lifetime have you ever used marijuana (pot, weed, grass)?”; *Inhalants*: “In your lifetime have you ever sniffed glue, breathed the contents of an aerosol spray can, or inhaled other gases or sprays, in order to get high?”; *Other illicit substances*: “In your lifetime have you ever used other illegal drugs (like cocaine, heroin, ecstasy, or amphetamines/speed)?”. Participants responses to the lifetime substance use questions were dichotomised (*0* “never used”, *1* “One or more times”).

#### Depressed mood

For the 2006 and 2017 surveys, the Short Mood and Feelings Questionnaire (SMFQ; Angold et al., 1995) was used. This is a 13-item measure of mood over the past 2 weeks (alpha = 0.91). Example items include “I felt miserable or unhappy” and “I didn’t enjoy anything at all”, with responses ranging from 1 (*“Not true”*) to 3 (*“True”*). Higher scores indicate a greater level of depressive symptoms (scores range from 0 to 26). For the 2009 survey, the Kessler Psychological Distress Scale (K10) (Kessler et al., 2002) was used. Each of 10 items (e.g., “In the past 4 weeks, about how often did you feel hopeless/nervous/worthless?”) is rated using a 5-point Likert scale (*1 “*none”, *2* “a little”, *3 “s*ome”, *4* “most”, *5* “all of the time”). K10 scores range from 10–50 with higher indicating greater levels of psychological distress in the past 4 weeks. The internal reliability of the K10 for the present data set was excellent (Cronbach’s = 0.90).

#### Emotional control

Emotional control was measured using four items on a four-point scale (*1* “definitely yes”, *2* “yes”, *3 “*no”, *4* “definitely No”). The four items were “I know how to relax when I feel tense”, “I am always able to keep my feelings under control”, “I know how to calm down if I am feeling nervous”, and “I control my temper when people are angry with me” (Cronbach’s Alphas range 0.74–0.79).

#### Academic problems

Academic failure was measured using 2 items: “Putting them all together, what were your marks like last year?” (5-point scale from *1* “very good” to *5* “very poor”), and “Are your school marks better than the marks of most students in your class?” (4-point scale from *1* “definitely yes” to *4* “definitely no”). Opportunities for prosocial involvement in school was measured using five items (e.g., “In my school, students have lots of chances to help decide things like class activities and rules”, “There are lots of chances for students in my school to talk with a teacher one-on-one”) (Alphas 0.57 to 0.65 across surveys).

#### Family variables

Poor family management was measured using 9 items on a 4-point scale (*1* “definitely yes” to *4* “definitely no”). Example items include “My parents ask if I’ve done my homework”, “Would your parents know if you did not come home on time”, “The rules in my family are clear” (Alphas ranged from .84 to .85 across surveys). Family conflict was measure using 3 items on a 4-point scale (*1* “definitely yes” to *4* “definitely no”): “We argue about the same things in my family over and over”, “People in my family have serious arguments”, and “People in my family often insult and yell at each other” (Alphas ranged from .78 to .83 across surveys). Parents’ favourable attitude to substance use was measure using 4 items (*1* “very wrong”, *2* “wrong”, *3* “A little bit wrong”, *4* “Not wrong at all”): “How wrong do your parents feel it would be for you to … ” “smoke cigarettes”, “drink beer or wine regularly (at least once or twice a month)”, “drink spirits regularly? (at least once or twice a month)”, and “use marijuana (pot, weed, grass)” (Alphas ranged from 0.78 to 0.82 across surveys).

### Analyses

Statistical analyses were conducted using SPSS (Version 25) for the logistic regression and Mplus version 8.1 (Muthen & Muthen, 1998 − 2017) for the latent class analysis. To estimate school level effects in substance use, intra-class correlations were calculated for the three surveys and for the five substance use categories. Thirteen of fifteen ICCs were below the value where multilevel or mixed models are recommended (.05; Hedges & Hedberg, 2007) and the ICCs for alcohol use and tobacco use were marginally over 0.05 for the 2006 survey only (.07 and .06 respectively), so school level effects were not modelled in subsequent analyses.

Latent class analyses were performed, respectively, on all three surveys using the lifetime substance use variables: Tobacco, alcohol, marijuana, inhalant, and other illicit substance use. Fit indices included the Akaike Information criteria (AIC; Akaike, 1987), the Bayesian Information Criteria (BIC; Schwarz, 1978) and the Sample Size-adjusted Bayesian Information Criteria (Sclove, 1987). Entropy values estimated on the average posterior probabilities were used to evaluate class quality, with higher values signalling clear class separation (Muthén, 2004; Nagin, 2005). Model fit statistics were conducted beginning with a 2-class solution and sequentially tested up to 4 classes. To evaluate predictors of cluster membership, stepwise logistic regression (SPSS Version 25) was used. Independent predictors were grouped by individual predictors (Block 1: depressed mood, emotional control), family predictors (Block 2: poor family management, parent’s attitude to substance use), and school predictors (Block 3: academic failure, opportunities for prosocial involvement at school). School level clustering of data was not modelled because of the extreme zero-inflatedness of polydrug class counts.

## Results

### Latent class analyses

**Figure 1.**
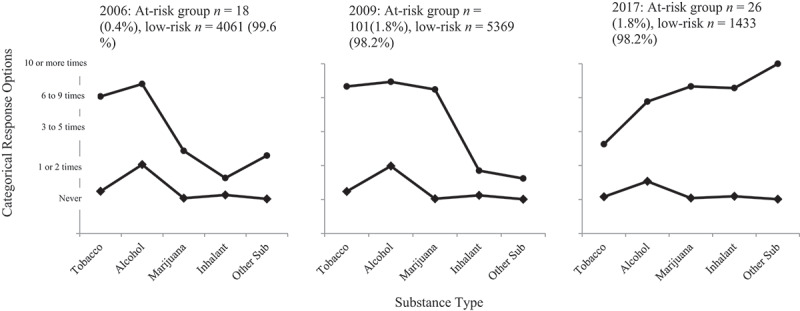
Estimated means for lifetime substance use across the three surveys.

*Notes*. The same five response categories were used for each lifetime substance use question across the three datasets: 1 = “Never”, 2 = “1 or 2 times”, 3 = “3 to 5 times”, 4 = “6 to 9 times”, 5 = “10 or more times”. -♦- = low-risk class; -●- = at-risk class. Other Sub = Other substance use (cocaine, heroin, ecstasy, or amphetamines/speed).

Figure 1 depicts the estimated means for lifetime substance use associated with each class across the three surveys.

#### 2006 survey

The 2-class and 3-class solutions had the greatest entropy values (both values of >.99), but the 3-class solution was not viable as it included a class with a single member. A 2-class model was chosen as the optimal solution as it yielded classification that was clearly distinct and interpretable, had high average posterior probabilities signalling clear class separation, and subgroup sizes which presented as more interpretable for making meaningful comparisons between the three years (see Table 1). *Class 1* participants (the ‘at-risk class) were the minority (<1%, *n* = 18) and were predominantly high frequency tobacco and alcohol users and low frequency cannabis and other illicit drug users (see Figure 1). *Class 2* participants (99.6%; *n* = 4061) were predominantly low frequency alcohol users and reported almost nil use of other substances. This class was labelled the *low-risk* group and designated the reference group in regression analyses.

**Table 1.** Fit indices from latent class analyses for the three surveys.

	2006			2009			2017		
	2-class	3-class	4-class	2-class	3-class	4-class	2-class	3-class	4-class
SSBIC	13566.88	8410.58	3815.22	35687.46	28820.20	22086.81	6376.148	5001.58	3972.54
AIC	13516.70	8341.58	3727.41	35632.59	28744.76	21990.79	6342.41	4955.19	3913.49
BIC	13617.72	8480.48	3904.19	35738.31	28890.11	22175.80	6426.98	5071.47	4061.48
Entropy	1.00	1.00	0.99	0.99	0.99	0.99	0.99	0.99	0.98
Subgroup size									
C1	18	17	17	101	279	281	26	34	16
C2	4061	4061	3878	5369	23	22	1433	22	81
C3		1	1		5168	77		1403	144
C4			183			5090			1218

SSBIC = Sample size adjusted Bayesian information criteria. AIC = Akaike information criteria. BIC = Bayesian information criteria. C1 = Class 1; C2 = Class 2; C3 = Class 3; C4 = Class 4.

#### 2009 survey

The optimal solution was a two-class solution as it yielded classification that was clearly distinct and had strong interpretability and high average posterior probabilities (see Table 1). *Class 1* participants (“at-risk” class) were the minority (1.8%, *n* = 101) and were predominantly high frequency users of tobacco, alcohol, and marijuana users, and lower frequency users of inhalants and other substance users (see Figure 1). *Class 2* participants were predominantly low frequency alcohol users and the use of other kinds of substances was rare. This class was labelled the low-risk class and was used as the reference group (98.2%; *n* = 5369).

#### 2017 survey

As for the prior surveys, a 2-class model had the highest entropy, interpretability, and average posterior probabilities (see Table 1). *Class 1* participants (“at-risk” class) were the minority (1.8%, *n* = 26) and were predominantly high frequency alcohol, cannabis, inhalant and other substances users (see Figure 1). They also reported elevated tobacco use. *Class 2 (“low risk”)* participants were predominantly low frequency alcohol users and used almost no other kinds of substances (98.2%; *n* = 1433).

### Regression analysis

We limited our regression analyses (dependent variable class membership) to the 2009 survey, where the absolute number of polydrug users was highest ^1^. The results of the logistic regression are presented in Table 2 and summarised here. In Block 1, emotional control but not psychological distress was significantly associated with membership of the *at-risk* class. When family predictors were entered (Block 2), family conflict (*p* = .063), poor family management, and parents’ attitude to substance use predicted the “at-risk” class membership and emotional control was retained as a significant predictor. When school predictors were entered (Block 3), the following predictors were significant: academic failure, poor family management, and emotional control, and there was a near-significant trend for family conflict (*p* = .082). Psychological distress, parental attitudes to substance use, and opportunities for prosocial involvement (*p* = .381) were not significant predictors of class membership.

**Table 2.** Logistic regression results showing the prediction of at-risk class membership relative to low-risk class membership.

Variables	*B*	95% CI	R^2^
Individual predictors (Block 1)			.066
Emotional Control	.29***	[1.19,1.49]	
Psychological Distress	.01	[.97,1.04]	
Family predictors (Block 2)			.184
Emotional Control	.15*	[1.03,1.30]	
Psychological Distress	−.02	[.95,1.02]	
Poor Family Management	.15***	[1.10,1.22]	
Family Conflict	−.11‡	[.80,1.01]	
Parents’ Attitude to Substance Use	.10*	[1.01,1.20]	
School predictors (Block 3)			.201
Emotional Control	.13*	[1.01,1.28]	
Psychological Distress	−.02	[.95,1.02]	
Poor Family Management	.14***	[1.09,1.22]	
Family Conflict	−.10†	[.80,1.01]	
Parents’ Attitude to Substance Use	.09*	[1.00,1.19]	
Academic Failure	.29**	[1.10,1.62]	
Opportunities for Prosocial Involvement at School	.06	[.92,1.20]	

^†^*p* = .082, ^‡^*p* = .063, *p < .050, **p < .010, ***p < .001. Higher scores on emotional control reflect poor abilities to manage one’s emotions. Higher scores on parents’ attitude to substance use indicates more favourable attitudes related to the offspring’s substance use. Higher scores on the family conflict scale represent lower family conflict.

## Discussion

This study investigated profiles of lifetime substance use across three population surveys, and the association of individual, familial and school factors with these latent classes. The great majority of adolescents aged 12–14 years reported almost nil substance use. Each survey had a small but notable minority (between .44% and 1.85%) of adolescents reporting the use of multiple substances (labelled polydrug users). The topography of substance use showed meaningful variations across the three surveys. In 2006, polydrug users reported elevated frequencies of tobacco, alcohol, and cannabis use, and one or two instances of inhalant and other (illicit) substance use. In 2009, polydrug users reported elevated frequencies of tobacco and alcohol (similar rates to 2006) and more frequent cannabis use than 2006. Inhalant use and other substance use appeared similar to 2006 rates. In 2017, polydrug users reported lower frequencies of tobacco use compared to 2006, similar levels of alcohol and cannabis use, and higher frequencies of inhalant and other (illicit) substance use. Compared to the low-risk group, polydrug users reported poorer family management practices and greater problems with emotional control. The results are consistent with the possibility that adolescents are at elevated risk of polydrug use when parental awareness and supervision of adolescent activities are poor and adolescents have difficulties managing associated negative emotions. Polydrug use may be used instrumentally to control negative emotions and engaging with peer networks that use multiple drugs may be a way of escaping distressed and disengaged families.

The findings on lifetime prevalence of polydrug use in these Australian 12–14 year old surveys were comparable to those found in Brazil (1.8%; Valente et al., 2017) and Australian nationally representative studies (2%; White et al., 2013), but below the prevalence rates of polydrug use reported in other countries, including the United States (Cranford et al., 2013). The present prevalence rate was higher than prior research in Thailand (0.6%; Assanangkornchai et al., 2018) but this was unsurprising because their classes included a range of high-risk behaviours additional to substance use (e.g., carrying a weapon), which is rarely reported in Australian early adolescent populations. The similarity of findings from these surveys and prior National Drug Strategy Household Survey data points to the convergent validity of the present study. It is also notable that the findings were relatively consistent across informed consent mechanisms, suggesting that downward biases associated with active parental consent mechanism were minimal: The 2009 survey (which used passive parental consent) had slightly higher polydrug use prevalence rates than both the 2006 (by 1.41%) and the 2017 survey (by 0.07%) (both of which used active parental consent). However, the magnitude of these variations was small given that active parental consent mechanisms were associated with the exclusion of 27.5% of the field of potential participants.

There are significant challenges with detection and intervention for polydrug users, given the low prevalence rate for this class. Interventions for polydrug use might follow a stepped care approach (Toumbourou et al., 2021), where students experiencing problems are screened for drug use and targeted individual interventions are delivered by school-based health professionals. Evidence-based interventions for polydrug using early adolescents might require a comprehensive focus on emotional control problems, strengthening school achievement, and screening/intervention for a variety of substances. Intensive and sustained multimodal programs addressing academic performance through tutoring, drug use prevention, and family risk factors are effective (Conduct Problems Prevention Research Group, 2004). Longitudinal research findings and controlled trials point to the value of family-oriented interventions for addressing adolescent depression and drug problems and this is consistent with the family risks established in the present study (Chan et al., 2013; Mason et al., 2003, 2012). Longitudinal research would help to establish dominant paths of influence, which in turn would guide the relative weighting program components and delivery modes.

A strength of this study is that it utilised three large-scale Australian samples, it included a mix of pathways to participation permitting redress of potential sample bias (Kelly & Halford, 2007), and it focused on young adolescents (12–14 year olds) where data is scarce. The study is cross-sectional and so causal relationships cannot be established. The cell sizes for polydrug users were generally small and necessitated a focus on key variables at the exclusion of other potentially important variables. Substance use and problems amongst other family members were not assessed as part of this study, and it is possible that these factors may be significant drivers of adolescent polydrug use. We did not include sex and age in our analyses because cell sizes for polydrug users were small so it was necessary to minimise the number of independent variables entered into each model. Also, there were no meaningful differences in sex and age across the three surveys, there was an almost even split of genders across the surveys, and the age range for inclusion in the study (12–14 years of age) was small.

## Conclusion

This study showed that a small proportion of 12–14-year-old adolescents use multiple drugs and that this prevalence has changed very little over the last 14 years, despite great population-level success in the reduction of specific drug use, including tobacco and alcohol. Depressed mood and emotional control problems characterise polydrug users. Tobacco use was less prominent in the drug use profiles of the most recent survey. The findings point to the importance of improving early detection of at-risk adolescents and providing interventions that are tailored to meet complex presentations.

## Supplementary Material

Supplemental Material

## Data Availability

The data that support the findings of this study are available from the last author, Prof. John Toumbourou, Deakin University, john.toumbourou@deakin.edu.au, upon reasonable request.

## Note

1. We elected not to include the findings of the logistic regression for the 2006 survey and the 2017 survey because of the very small cell sizes of polydrug users (*n* = 18 and 26 for the 2006 and 2017 survey respectively) and the relatively large number of variables in the full model (7 independent variables). For completeness we note that for the 2006 dataset the results for the same model were largely consistent with the 2009 data set, with significance for emotional control, poor family management and parental attitudes towards substance use, and nonsignificance for depressed mood. For the 2017 survey, none of the variables were significant. The most likely account of the pattern of nonsignificance for the 2017 survey compared to the 2009 survey is lack of statistical power.
