# Supplementary material for: Polydrug use in Australian 12-14 year olds from 2006 to 2017: an examination of drug use profiles, emotional control problems, and family relationship characteristics
Source: Aust J Psychol. 2023 Feb 19;75(1):2174705. doi: 10.1080/00049530.2023.2174705 (PMC12175681; doi:10.1080/00049530.2023.2174705)
Supplement: Supplemental Material [file RAUP_A_2174705_SM2412.docx]

Table S1. *Summary of study characteristics, methodology, and results of studies employing latent class analysis of drug use patterns among adolescents*

| **Author(s) (year)** | **Sample size** | **Sample characteristics** | **Sample represent** | **Drug types in LCA** | **Definition of polydrug use** | **Key drug use measures** | **Cluster names and prevalence** | **Cluster descriptors** | **Measures of academic performance and school factors** | **Correlates of academic performance with polydrug use findings** |
| --- | --- | --- | --- | --- | --- | --- | --- | --- | --- | --- |
| Assanangkornchai et al. (2018) | *n* = 25566 | 54% female  Age range = 12-15 years  Location: Thailand | Sample drawn from 119 schools using 2-stage stratified clustering, responses from the National School survey. | A C E H IN MT T  Cannabis Anxiolytics Meth  Ketamine  Kratom  Opium | Past month A use; past year drug use | 20 discrete variables assessing different health risk areas (A use, drugs, behaviours related to unintentional & intentional injury) | 3 clusters - I. Low risk behaviours (88%); II. Moderate risk behaviours (11%); III. High risk behaviours (0.6%) | I. low-risk behaviours (moderate A use) II. Moderate-risk behaviours e.g., driving under the influence of A, fighting, carrying a weapon, A & T III. High-risk behaviours e.g., use of illicit drugs (kratom & cannabis) | GPA (0 - 4, with higher indicating better performance). | Moderate & High-risk classes associated with a lower GPA |
| Banks et al. (2020) | White:  *n* = 7271  Black:  *n* = 1301 | White: 54.61% female  Black: 59.89% female  Age nr.  Location: USA (midwest) | State sample of 159 schools from 21 districts, drawn from a larger longitudinal study.  Grades: 9-12 | T A M I  Other (e.g., C E LSD MT) | Past 30-day use | Frequency of use in past 30 days (0 days/1-2/3-9/10-30) | Black: 4 clusters - I. Non-use (87.8%); II. A & M use (6.3%); III.A, M, & cigarette use (3.8%); IV. Frequent Polysubstance use (2%)  White: 5 clusters - I. Non-use (73.4%); II. A (13.9%); III. A, M, & cigarette use (9.4%); IV. Moderate Polysubstance use (1.6%); Frequent Polysubstance use (1.7%) | Black: I. Low prob. of any substance use in the past 30 days II. High prob. of M & A use only III. High prob. of A, M, & T (cigarette use) only  IV. High prob. use of all substances  White: I. Low prob. of any substance use in the past 30 days II. High prob. of A use only III. High prob. of A, M, & T (cigarette use) only  IV. Moderate Polysubstance use  V. Frequent Polysubstance use |  |  |
| Chan et al. (2016) | *n* = 5412 | 50% male; *M*age = 13.50  50% female; *M*age = 13.43 Location: Australia (Victoria) | Participants randomly selected via 2-stage cluster sampling. Grades: 7, 9, 11 | A T C IN Other | Past 30-day use | Frequency of use in past 30 days (0 times/1-2/3-5/6+) | 3 clusters - I. No drug use (68.2% male, 69.14% female); II. Mainly A use (26.02% male, 26.68% female); III. Polysubstance use (5.06% male; 4.18% female) | I. No substance use II. Mainly A use III. Very high prob. using A & T, moderate prob. using C, small prob. using IN & other illicit drugs. | School connectedness (control variable) Academic failure (control variable) |  |
| Chan et al. (2017) | *n* = 9966 | 49.34% male  *M*age = 14.3 Location: Australia (Victoria) | Participants randomly selected via 2-stage cluster sampling. Grades: 7, 9, 11 | A T C IN Other | Past 30-day use | Frequency of use in past 30 days (0 times/1-2/3-5/6+) | 3 clusters - I. Non-use (47.7%); II. A use (44.1%); III. Polysubstance use (8.2%) | I. No substance use II. Mainly A use III. Very high prob. using A & T, moderate prob. using C, small prob. using IN & other illicit drugs. | School commitment (7 items); Reward for prosocial school involvement (3 items); Academic failure (2 items) | Low school commitment & academic failure related to III. (compared to I.); academic failure associated with III. (relative to II.). High school commitment & prosocial involvement reward related to lower likelihood of polysubstance use. |
| Charak et al. (2015) | *n* = 918 | 49.6% female; *M*age = 14.9; 68.3% Caucasian Location: USA | Participants reporting sexual/physical assault selected from a larger study (*N* = 4023) | T A M C PCP LSD H MD IN NUPM | Lifetime use | Ever use of T & A; frequency of T (cigarettes / snuff / chewing) in past 30 days; frequency of A & heavy drinking in past year (5+ drinks one occasion); ever use of non-prescribed or illicit drugs (recoded as single drug use or use of 2+ drugs). | 3 clusters - I. Experimental use (35.95%); II. Light polysubstance use (35.62%); III. Polysubstance use (28.43%). | I. Minimal use II. High prob. heavy drinking in past year, use of NUPM, single type drug use  III. Regular use T, heavy drinking, NUPM, single- & poly-drug use |  |  |
| Connell et al. (2010) | *n* = 1236 | 53% female; *M*age = 14.6; 89% Caucasian  Location: USA (Connecticut) | Regional sample representing 10 public/non-vocational schools.  Grades: 9-10 | A T M NUPM IN C H E | Past 30-day use (illicit drugs); lifetime use (NUPM; IN) | Lifetime use & past 30-day use (frequency of use on an 8-point scale, from no use to daily use). These 2 measures combined into 4-point scale: 1) no history of use; 2) no 30-day past use; 3) use 1-5 days; 4) use 6+ days. | 4 clusters - I. Non-users (22%); II. A experimenters (38%); III. Occasional polysubstance users (29%); IV. Frequent polysubstance users (10%) | I. No history of substance use II. Moderate prob. of lifetime A use; lower prob. of A use in past month III. Higher prob. of use for each substance than I. or II; many with history of A, T, M use although low prob. of use in past month. IV. Highest prob. of initiation for each substance; past month use > 6 or more days high for A, T, & M; highest prob. lifetime hard drug, IN, or NUPM use | Academic grades (self-reported 9-point scale - mostly As to mostly Fs - converted to a score from 0–4 to reflect approximate GPA) Youth commitment to school & education (self-reported 5-items on a 4-point scale) | Higher grades associated with reduced odds of frequent/occasional polysubstance use (relative to experimental alcohol use or non-use) |
| Cranford et al. (2013) | *n* = 2744 | 50.4% female; *M*age = 14.8; 64.1% Caucasian  Location: USA (Michigan) | Internet survey of adolescents across 2 districts representing 5 schools. | T A M I NUPM EXPM | Past 12-months use | Frequency of use in past 12 months - NUPM (≥1 of 6 types), EXPM (≥1 of 7 types), T, A, M, Illicit drugs, Internalizing/externalizing problems - Youth Self-Report (YSR; 112 items; Achenbach & Rescorla, 2001) Parental monitoring (6 items); Children of Alcoholics Screening Test (CAST; 6 item version; Jones, 1983). | 4 clusters - I. Low/no use (76.3%); II. Multiple illicit drugs (4.2%); III. TAM (11.5%); IV. NUPM, EXPM (8.0%) | I. No / very low prob. using any substances (past 12 months) II. Relatively high prob. using all substances ≥1 time (past 12 months) III. Relatively high prob. using three of the six substances (TAM) ≥1 time (past 12 months) IV. Relatively high prob. A, NUPM, & EXPM (past 12 months) |  |  |
| Delk et al. (2019) | *n* = 2733 7th grade: *n* = 765 9th grade: *n* = 913 11th grade: *n* = 1055 | 7th grade: 48.8% female, *M*age = 12.3; 53.8% Hispanic 9th grade: 48.9% female, *M*age = 14.3; 55.7% Hispanic 11th grade: 49.0% female, *M*age = 16.0; 52.8% Hispanic Location: USA (Texas) | Drawn from Wave 3 of the Texas Adolescent Tobacco & Marketing Surveillance System (*N* = 461069), 79 schools represented | T A M | Past 30 days use (current use) | Ever use, current use, & susceptibility to T (eCigarettes & combustible) Current use of A & M BD (past 14 days) | 7th grade: 2 clusters - I. No risk (77.5%); II. Tobacco susceptible (22.4%) 9th grade: 3 clusters - I. No risk (48.3%); II. Tobacco susceptible (37.8%); III. Tobacco ever use (13.9%) 11th grade: 4 clusters - I. No risk (39.5%); II. Tobacco susceptible (26.1%); III. Tobacco ever use (24.4%); IV. All products use (10%) | I. low prob. of susceptibility to or use of products.  II. high prob. of being susceptible to combustible T III. high prob. of ever use of combustible T IV. high prob. of ever use of combustible T & e-cigarettes, high prob. past 30-day A & M use, past 14-day BD |  |  |
| Göbel et al. (2016) | *n* = 33566 | 50.6% female; *M* age = 13.9 Locations: Armenia, Sweden, Austria, Belgium, Bosnia & Herzegovina, Cyprus, Czech Republic, Denmark, Estonia, Finland, France, Germany, Hungary, Iceland, Ireland, Italy, Lithuania, The Netherlands, Switzerland, Norway, Poland, Portugal, Russia, Slovenia, Spain | Data from the Second International Self-Report Delinquency Study (ISRD-2), administered in 25 European countries (*N* = 67883). Participating schools randomly selected. Grades: 7-9 | A M  Other Illicit substances | Past month use of other illicits, frequency of recent use | Lifetime use, recent use (past month), & frequency of recent use (0 times, 1-2, 3+), for 4 categories of drug: A (beer, wine, alcopops); Spirits (e.g., rum, vodka, whisky); M; Other illicit substances (e.g., E AM LSD H C) | 4 clusters - I. Non-users (68%), II. Low A users (16.1%), III. A users (11.2%); IV. Polysubstance users (past month) (4.7%). | I. Non-users II. Recent use of A (e.g., beer, wine, alcopops) III. Recent use of A; lifetime use of M IV. Recent use of A, M, & other illicit drugs |  |  |
| Karlsson et al. (2019) | *n* = 3374 | 56.1% male  Age nr Location: Sweden | Selected from a nationally representative larger survey (*N* = 36007) if they reported illicit drug lifetime use Grades: 9, 11 | M AM C E H Spice Hash | Combination of past 30 days use; past 12 months; 10+ times | 10 binary questions: Use of illicit substances in past 30 days and in past 12 months, & 10+ times. Respondents indicating 'yes' to any of these items asked to indicate use of 7 specific drugs. | 4 clusters - I. M testers (60%); II. M users (17%); III. Cannabinoid users (15%); IV. Polydrug users (7%) | I. lowest prob. illicit substance use of all classes (low prob. of 10+ times use); infrequent use of M II. Highest prob. use in past 30 days & 12 months; less likely to have tried other 6 drugs III. High prob. use HA, M, SP. Highest prob. drug use 10+ times; lower prob. use in past 30 days IV. High. prob on all indicators; very likely to have used 10+ times |  |  |
| Kelly et al. (2015) | *n* = 10273 | 49.34% male *M*age = 12.51 years (Grade 7); 14.46 years (Grade 9); 16.4 years (Grade 11) Location: Australia (Victoria) | Participants randomly selected via 2-stage cluster sampling. Grades: 7, 9, 11 | A T C IN Other | Past 30-day use | Frequency of use in past 30 days (0 days; 1-2; 3-9; 10-30) | 3 clusters - I. No drug use (47.7%); II. mainly A use (44.1%); III. Polydrug use (8.2%). | I. 0 prob. use any drugs (specified *a priori*) II. Moderate to high prob. use of A, small prob. use of T (other drug use negligible) III. High prob. use of A & T, moderate prob. use M, small prob. use other illicit substances | Academic failure (2 items) History of suspension from school (1 item; binary response) | Polydrug use (relative to mainly A use class) positively associated with academic failure & school suspension |
| Miech et al. (2016) | *n* = 16615  Grade 8: *n* = 5060  Grade 10: *n* = 4443  Grade 12: *n* = 8597 | Grade 8: 51.25% female  Grade 10: 50.06% female  Grade 12: 51.17% female  Age nr Location: USA | Data drawn from Monitoring the Future study (*N* = 41551) using a multistage stratified approach, nationally representative.  Grades 8, 10, 12 | eC T M/Hash NUPM BD | Past 30 days use (past 2 weeks for BD) | Past 30-day drug use (binary questions), or past two weeks for BD episodes (defined as 5+ drinks in a row) | Grade 8: 2 clusters - I. Low-level users (91%); II. Poly-users (9%) Grade 10: 2 clusters - I. Low-level users (82%); II. Poly-users (18%) Grade 12: 3 clusters - I. Low-level users (76%); II. Predominant eC users (7%); III. Poly-users (17%) | Grade 8:  I. Low prob. of substance use, eC use highest prob. use (3x as high as any other substances) II. High prob to use any of the 5 substances (slightly lower for NUPM)  Grade 10: I. Similarly low prob. use of eC, M, & BD II. High prob to use any of the 5 substances (eC more prominent) Grade 12: I. Low prob. of substance use II. Very high prob. eC use, moderate prob. M, BD, & T use III. High prob to use any of the 5 substances (eC more prominent) |  |  |
| Nakawaki & Crano (2015) | *n* = 7476 | 53% female  Age range = 12-17 Location: USA | Sample drawn from 2012 National Survey of Drug Use & Health (NSDUH), if they reported past year IN use | T A M IN C H Hallucinogens NUPM (Opioid, Sedative, Stimulant, Tranquiliser) | Past year users & non-past year users | Past year use (binary questions) for each of the 8 substances | 6 clusters - I. high substance use/high delinquency (5.6%); II. cigarettes/alcohol/ M /opioids /moderate delinquency (11.7%); III. low substance use/fighting (17.5%); IV. high substance use/low delinquency (10.1%); V. Cigarette/A/M (23.4%); VI. low substance use/low delinquency (31.8%) | I. high in both substance use & delinquency II. high prob. of T, A, & M use, moderately high nonmedical use of prescription opioids, & moderate levels of delinquency.  III. past year fights (both alone & in groups), low prob. of substance use & delinquency.  IV. high prob. substance use & low delinquency.  V. likely to use IN, cigarettes, A, & M; otherwise, low substance use & delinquency.  VI. low prob. substance use & delinquent behaviours. | Academic grades during most recent grading period on 4-point scale ('A' to 'D or less') | Poorer grades in Class 1 - 5 (relative to Class 6) |
| Schnieder et al. (2020) | *n* = 387 | 60% female  **Age nr**  77% non-Hispanic Black Location: USA (Maryland) | Selected from a larger sample (*N* = 805) if they reported lifetime use ≥1 drug Grades: 9-12 | A M SM C MT H PM IN E ST | Lifetime use | Lifetime use of 10 drug types | 3 clusters - I. A & M use (68.6%); II. Polysubstance use (22.0%); III. A/PM/IN use (9.4%) | I. High prob. use A & M II. High prob. use A & all drug types III. A, IN, & PM use, some M use. |  |  |
| Valente et al. (2017) | *n* = 6381 | 51.21% female  *M* age = 12.61 Location: Brazil | Baseline data from a randomised control trial testing a school-based substance use prevention program in 6 cities.  Grades: 7-8 | A BD T M IN | Past 12-month use | Past 12-month use of drug types (includes BD episodes, defined as 5+ drinks in 2hr period) | 3 clusters - I. Abstainers/low users (81.54%); II. A use / BD (16.65%); III. Polydrug users (1.80%). | I. Average prob. A use, very low prob. other drug use. 0 BD episodes II. High prob. A & BD, average prob. IN, low prob. T, very low prob. M III. High prob. use of all drugs |  |  |

*Note.* nr = not reported, T = tobacco, A = alcohol, BD = binge drinking; M = marijuana*,* SM = synthetic marijuana, C = cocaine; PM = pain medications (non-prescribed); ST = steroids; IN = inhalants; E = ecstasy; H = heroin; LSD = Lysergic acid diethylamide; MT = methamphetamine; MD = methadone; AM = amphetamine; PCP = phencyclidine; NUPM = nonmedical use of prescription medications; EXPM = excessive use of prescription medications; eC = eCigarette; prob. = probabilities
